# Supplementary material for: Are carbon emissions trading and green financial instruments synergistic? -Comprehensive quantitative research based on content analysis
Source: PLoS One. 2024 Mar 7;19(3):e0298601. doi: 10.1371/journal.pone.0298601 (PMC10919601; doi:10.1371/journal.pone.0298601)
Supplement: S1 Data — (ZIP) [file pone.0298601.s001.zip › Data/地级市Z_F工作报告.zip!/┴╔─■/╔≥╤⌠╩╨/└┤╫╘╣1⁄2╓┌║┼í╛┬φ┐╦╩2╛▌═°í┐_2017.docx]

各位代表：

　　现在，我代表市政府向大会报告工作，请予审议，并请市政协各位委员提出意见。

　　一、2016年主要工作回顾

　　过去一年，在市委的坚强领导下，在市人大及其常委会依法监督和市政协民主监督下，市政府紧紧依靠全市人民，全面贯彻党的十八大、十八届三中、四中、五中、六中全会和习近平总书记系列重要讲话精神，按照五大发展理念和“四个着力”要求，积极推进中央新一轮东北振兴战略实施，认真落实市第十三次党代会部署，坚持稳中求进工作总基调，以供给侧结构性改革为主线，统筹抓好各项工作，努力推动全市经济社会平稳发展。

　　预计全市地区生产总值完成6712亿元；固定资产投资完成1621亿元；一般公共预算收入完成620.9亿元，增长2.4%；社会消费品零售总额完成3960亿元；城镇居民人均可支配收入达到39135元，增长6.8%；农村居民人均可支配收入达到14445元，增长7.1%；城镇登记失业率为3.12%。

　　(一)认真落实新一轮振兴战略，努力推进供给侧结构性改革，经济发展质量效益明显提高

　　积极落实供给侧结构性改革重点任务，出台稳增长政策措施，全市经济逐步企稳向好。地方税收占一般公共预算收入比重达到82.4%，提高1.2个百分点。

　　振兴政策举措有效实施。认真贯彻中央关于新一轮东北振兴决策部署，系统研究和编制沈阳振兴发展战略规划，明确振兴发展总体思路和行动方案。对接《推进东北地区等老工业基地振兴三年滚动实施方案》，细化分解任务，狠抓工作落实。主动开展项目、政策、资金对接，争取到国家和省重大项目162个、政策85项、资金251.1亿元，政策红利得到有效释放。

　　“三去一降一补”成效明显。积极支持企业“走出去”化解产能，新签对外投资超千万美元项目15个、协议投资额11.3亿美元，联立铜业入选“2016年中国企业全球化新锐50强”。出台房地产新政33条，全市商品住房备案面积增长9.8%，去化周期缩短到7个月。采取债券置换、政府与社会资本合作等措施，政府性债务风险得到有效防控。全面实施“营改增”和降本减负20条政策，减免行政事业性和经营服务性收费55项，为企业和居民直接减负约55亿元。

　　项目带动战略扎实推进。出台壮大战略性新兴产业、差别化供地等政策措施，开工建设一批调结构、补短板重大项目。335个重点产业项目完成投资962.4亿元，132个政府投资项目完成投资142亿元，城市地下综合管廊等7个政府与社会资本合作项目启动实施，有效投资规模持续扩大。

　　(二)加快推进结构调整，努力建设制造强市、服务业大市，产业转型升级取得新进展

　　积极培育新产业、新业态、新模式，推动三次产业协调发展，服务业增加值占地区生产总值比重提高6.3个百分点。

　　工业加快转型升级。实施优势企业倍增计划和困难企业帮扶计划，工业运行质量得到提升，华晨汽车首次挺进中国企业百强。启动建设64个制造业智能升级示范项目，机床集团入选2016中国“新经济”十大优秀案例，i5智能机床产量增长1.2倍，我市成为“中国制造2025”试点示范城市。沈鼓集团研制的国内首台120万吨乙烯压缩机组投入运转，航天新光集团研制的氢气涡轮泵、中科院沈阳自动化所研制的深渊着陆器和无人潜水器，成功应用于我国“深空、深海”工程。新兴产业快速发展，机器人产业实现业务收入70亿元、增长27.3%，航空产业完成产值311亿元、增长7%。

　　服务业层次水平不断提升。辽宁振兴银行获批在沈筹建，全市新增中国银行辽宁分行总部等各类金融及金融服务机构40家，我市获准开展跨境人民币创新业务试点。沈阳国际物流港跻身首批国家级示范物流园区行列，我市成为国家现代物流创新发展试点市。全市限额以上单位网上商品零售额增长42.4%，浑南电商基地进入全国优秀电商园区20强。全年举办中国城市规划年会等各类展会380项，会展交易额增长10.2%。我市成为国家文化消费试点城市。沈北新区、浑南区成为国家全域旅游示范区创建单位，全市旅游总收入增长13.1%。

　　现代农业加快发展。积极调整农业结构，创建粮油作物高产示范区25个，“三品一标”认证面积达797万亩，康平县获批创建国家有机产品认证示范县，法库县被评为国家农产品质量安全县。沈阳东北冷鲜港进境肉类指定查验场通过国家验收，开辟了辽宁进口肉类商品新通道。开通“沈满俄”班列，填补了东北地区鲜活农产品出口国际铁路联运空白。土地确权登记、经营权流转等农村综合改革稳步推进。新民市获批全国农村产业融合发展试点示范县，苏家屯区成为国家产城融合示范区。辽中区正式挂牌，获批国家新型城镇化综合试点区。

　　(三)深入推进全面创新改革，努力构建开放发展新格局，发展动力活力不断增强

　　精心编制全面创新改革试验方案并获国务院批复，积极探索、先行先试，形成了中德装备园体制机制创新等4项可复制推广经验。

　　科技创新取得新突破。出台自主创新示范区建设三年行动计划和10项政策，全市新增高新技术企业139家，是上年的3.2倍。组建以新松为龙头的机器人产业创新联盟，新增8个国家级工程研究中心(工程实验室)，我市国家级创新平台数量进入全国第一集团。富创精密IC设备关键零部件、芯源微电子显影设备等重大科技专项通过国家验收。浑南区成为全国首批区域“双创”示范基地，三好街两岸青年创业园被评为“海峡两岸青年创业基地”，新增15家国家级众创空间，我市入选全国科技和金融结合、知识产权质押融资试点城市。

　　行政体制改革向纵深推进。加快转变政府职能，积极推进“放管服”改革，取消下放108项行政职权，行政审批事项削减46%、要件削减31.5%。在中德装备园等14个经济园区开展行政事业“零收费”试点，在大东区开展金融机构代办工商注册试点。公布并动态管理行政收费等6张目录清单，实施“五证合一、一照一码”，推行“双随机、一公开”监管，全市新增各类市场主体10.4万户、增长15.7%。

　　国资国企改革稳步实施。出台进一步深化国资国企改革实施意见和配套政策，旅游、养老、航空等7大产业集团投入运营，有效带动了优势资源整合与重点产业培育发展。市副食集团混合所有制改革取得新成效，机床集团履行出资人职责试点、东药集团内部用人制度改革试点等工作扎实推进，国有企业经营活力不断增强。

　　对外开放取得新成效。沈阳成为辽宁自由贸易试验区重点城市。成功接待德国总理默克尔来访，对德合作迈上新台阶。中德装备园集聚项目246个，成为中德智能制造合作试点示范园区。新开6条国际航线，与英国贝尔法斯特市结为友好城市，我市荣获“中国国际友好城市交流合作奖”。沈阳市跨境电商进口直购通关服务平台开通运行，我市成为全国服务外包示范市。主动融入“一带一路”，新签对外承包工程项目22个，北方重工集团获土耳其6台盾构机大单。境外合作园区建设加快，中德新松教育集团并购德国陶特洛夫职业培训学院，全市对外投资增长1倍。沈阳经济区一体化建设扎实推进，与营口港务集团共建的沈阳港集团投入运营。

　　(四)积极促进信息化与城市化融合，努力建设智慧城市，城市运行效率明显提高

　　深入落实国家“互联网+”战略，以“惠民、兴业、善政”为目标，积极打造智慧沈阳，我市获批国家大数据综合试验区，为城市转型发展奠定了坚实基础。

　　通信基础设施建设全面推进。开展“提速降费”行动，“光纤进小区”和行政村“光纤村村通”年度计划全面完成。无线网络四环内实现全覆盖，免费智能WiFi覆盖120个热点区域，全市公交车辆免费WiFi工程启动实施。我市成为“宽带中国”示范城市。

　　大数据产业加快培育发展。成功举办智慧城市(国际)创新大会。大数据发展生态体系不断完善，工业大数据创新发展试点有序推进，东网科技获2016中国大数据最佳创新企业奖。与浪潮集团合作推广“大数据创客模式”，大数据“双创”行动计划成效初显。华为、中兴、360、软通动力等60余家知名企业在沈投资兴业，以大数据为核心的新一代信息技术产业发展全面提速。

　　智慧城市应用体系加快建设。智慧沈阳统一平台上线试运行，数据共享交换平台、数据清洗融合平台和决策分析门户等功能投入应用，政府部门间数据交换和资源共享进程加快。政务协同办公平台投入运行，政府运行效率大幅提升、行政成本大幅下降。区域人口健康信息平台、智慧城市应急指挥中心等重点项目顺利推进，居民信息平台、智慧医保APP等投入使用，中小学全面建成“数字校园”，市属医院加快普及“智慧医院”，“乘车易”用户突破百万，“停车易、行车易”用户达到140万，城市运行和市民生活更加高效便捷。

　　(五)不断完善城市功能，努力打造生态宜居环境，城乡面貌持续改善

　　认真落实中央城市工作会议精神，坚持以人为核心，坚持建管并重，城市品质实现新提升。

　　基础设施建设扎实推进。地铁4、9、10号线和2号线北延线工程进展顺利，南北快速干道北段高架桥建成通车，五爱互通立交桥部分投入使用，百里二环实现无信号通行。新建改造城市慢行系统44万平方米，新增各类公共停车场47处、停车泊位9500个。投资15.7亿元加快宜居乡村建设。整修农村公路1810公里，为98个自然村通上柏油路。南运河、铁西新城、南北快速干道地下综合管廊开工建设，长青公园等8个“海绵城市”项目启动实施。农田水利设施不断完善，防洪减灾能力进一步增强。

　　生态环境明显改善。重拳治理雾霾，拆除10吨以下燃煤锅炉1917台，淘汰黄标车及老旧车辆5.6万辆，实施最严格的秸秆禁烧制度，强化工业排放、建筑工地扬尘管理，PM2.5、PM10浓度分别下降25%和18.3%，重污染天数比上年减少19天，大气优良天数增加42天。实施道路、水系、公园等重点区域绿化景观提升工程，全市绿化造林20.8万亩。百里环城水系综合治理、中小河流生态治理、污水处理厂建设和提标升级改造等工程加快推进，卧龙湖国家湖泊生态环境保护试点工作成效显著。

　　城市管理不断加强。构建以属地管理为核心的执法体系，数字城管加快向智慧城管升级，实行环卫常态化、标准化作业管理，市容秩序、市政设施、小区环境等12大专项治理行动全面启动。加快“公交都市”建设，新开调整公交线路27条，新增更新清洁能源公交车1327台，新增6条农村公交化客运线路，地铁实现常态化延时运营。推行城市生活垃圾分类管理，老虎冲渗沥液处理厂投入使用，城市垃圾“三化”处理能力不断提高。

　　(六)加大民生投入力度，努力建设幸福沈阳，发展成果更多惠及人民群众

　　实施十大民生工程，重点民生支出占财政支出比重达到85%，我市在联合国中国城市人类发展指数排名中居第四位。

　　就业和社会保障水平不断提高。成功举办“创响中国”巡回接力等“双创”活动，实施重点群体就业援助、企业稳岗补贴等政策，城镇新增就业8.4万人，城镇登记失业率低于控制目标0.88个百分点。企业退休人员基本养老金“十二连涨”，城乡低保“五连增”，城乡低保家庭冬季采暖救助实现全覆盖。我市成为全国首批生育保险和医疗保险合并实施试点城市。建成区域性居家养老服务中心17个，新增养老床位4042张，我市成为全国首批居家和社区养老服务改革试点城市。残疾人事业民生投入增长46.3%。11662个建档立卡贫困人口实现脱贫。投入3.84亿元实施城乡结合部老旧住宅“三网”配套改造工程，惠及居民7.43万户。完成棚改2.6万户(套)，基本建成保障房1.9万套，维修更新老旧住宅电梯212部，旧住宅区环境改造、农村危房改造、节能暖房改造等民生工程项目按计划完成。

　　公共服务供给能力持续提升。各级各类教育协调有序发展，增加公办幼儿园学位3500个，完成340所中小学旱厕、校舍改造，我市成为国家学前教育改革发展实验区、全国首批职业教育双元培育改革试点市。医疗综合服务能力显著提升，覆盖城乡的20个示范医联体全面建成，基层卫生服务体系建设进入全国示范行列，中医药事业加快发展，我市成为国家级医养结合试点城市。成功举办中国·沈阳国际合唱节，沈阳交响乐团受邀登上国家大剧院舞台，汗王宫遗址陈列馆、职工之家劳模纪念馆等文化设施建成开放，沈河区成为国家公共文化服务体系示范区。开展全民健身活动，有序开放各类全民健身场所。举办“十二强赛”、“哥德杯”世界青少年足球赛、沈阳马拉松等国际赛事，和平区世界足球公园、皇姑区气膜足球馆投入使用，“足球之都”建设为城市增添了活力。

　　社会治理创新取得新进展。加快和谐社区、智慧社区建设，积极推行“一口式受理、全科式服务、开放式办公”模式，社区服务信息化、智慧化、便利化水平不断提高。深化信访制度改革，及时发现和解决各类突出矛盾。公安“三大载体”活动深入实施，各类大型群众性活动安全有序，食品安全示范城市创建工作扎实推进，道路交通、消防、危险化学品等领域整治成效明显，安全生产形势和社会治安大局持续稳定。

　　(七)加快转变政府职能，努力加强政府自身建设，行政效能不断提升

　　按照打造国际化营商环境要求，积极推进政务环境建设，政府管理和服务水平不断提高。

　　法治建设持续推进。自觉接受人大法律监督、工作监督和政协民主监督，认真办理人大议案、代表建议和政协提案，提请市人大常委会审议法规草案4件，发布政府规章10件。出台法治政府建设实施方案和重大行政决策程序规定，建立重大决策合法性审查机制，进一步夯实了政府依法决策、依法行政的基础。

　　作风建设深入推进。迅速落实打造国际化营商环境意见，全面开展五大专项整治行动。推行公务员及时奖励制度，实施“督考合一”，激励约束机制更加完善。加强政府智库建设，拓宽群众建言献策渠道，切实增强了政府工作的针对性和实效性。

　　廉政建设不断加强。深入开展“两学一做”学习教育，认真落实中央巡视“回头看”整改任务，集中开展辽宁拉票贿选案警示教育，持续纠正“四风”，坚持厉行节约，“三公”经费支出继续压减。认真落实党风廉政建设主体责任，强化行政监察和审计监督，公务员队伍廉洁从政意识不断增强。

　　过去一年，国防动员、人防和民兵预备役建设进一步加强，我市实现全国双拥模范城“八连冠”。国家安全、保密、统计、参事、民族、宗教、侨务、贸促、广播电视、新闻出版、邮政、气象、防震减灾、档案、修志、文史、红十字会和慈善事业等工作都取得了新成绩。

　　各位代表，过去一年的成绩来之不易，这是市委正确领导的结果，是全市人民团结一心、共同奋斗的结果。在此，我代表市政府，向全市各族人民，向人大代表、政协委员、各民主党派、各人民团体和社会各界人士，向驻沈解放军、武警部队官兵和中省直单位，向关心支持沈阳振兴发展的港澳台同胞、海外侨胞和国际友人，表示诚挚的感谢和崇高的敬意！

　　回顾过去一年工作，我们也清醒地认识到发展中还存在一些问题。一是体制机制问题仍然突出，政府与市场关系尚未理顺，国资国企改革还不到位，民营经济发展还有很多制约，创新创业体系亟待完善。二是结构性问题仍然突出，重化工业比重过大，高附加值产品偏少，产业整体竞争力不强，县域经济相对薄弱，经济外向度不高，新旧动能还需加速接续转换。三是营商环境问题仍然突出，企业各类负担还比较重，投资吸引力下降，高层次人才外流，科研成果本地转化率不高。四是热点民生问题仍然突出，就业潜在压力不容忽视，城乡居民收入增长缓慢，公共服务水平还不高，环境污染、交通拥堵等“城市病”需要加大力度综合治理。五是政府职能错位、越位、缺位问题仍然存在，管理服务能力还不强，个别干部作风不实、担当意识不够，“放管服”改革还有很大的空间。

　　去年，我市地区生产总值、固定资产投资、规模以上工业增加值等指标未能完成预期目标，主要原因是，我们认真落实中央巡视组和国家审计署的反馈意见，举一反三抓整改，全面剔除虚增、做实经济数据，夯实后的指标与过去虚假数据存在不可比因素。同时也要看到，经济指标下降，表面上是外部需求不足，实质上是老工业基地长期以来积累的体制性、机制性、结构性矛盾的集中显现。

　　当前，外部环境依然严峻复杂，世界经济处于长周期衰退之中，不确定不稳定因素增多。我国区域经济走势分化，我市与先进地区在思想观念、工作方式、经济发展水平等方面差距拉大。经济增速下滑、结构调整阵痛、动能转换困难相互交织，经济运行风险增加，改革任务异常艰巨，振兴发展处在滚石上山、爬坡过坎的关键阶段。我们慢不得、更等不起，一定要增强责任感、紧迫感。同时，更要保持战略定力，坚定发展信心。

　　信心来自国家前所未有的政策支持。新一轮振兴战略深入实施，沈阳成为国家全面创新改革试验区、自主创新示范区和辽宁自由贸易试验区建设重点城市，赢得了多重历史新机遇。

　　信心来自发展基础和潜力。沈阳拥有雄厚的产业基础、较强的科研实力和一大批行业领军企业，区位优势日益明显，发展新动能正在不断集聚。

　　信心来自经济企稳向好的势头。过去一年，沈阳先行指标与全国同步增长，发展质量效益逐步提高，筑底企稳、回升向好的态势已经显现。

　　信心来自风清气正的政治生态。通过政治生态的修复，党风政风日益清朗清明，为沈阳振兴发展提供了坚强的政治保障。

　　信心来自干部群众的热切期盼。市第十三次党代会为沈阳振兴发展绘就了蓝图、指明了方向，全市上下已经汇聚起共谋振兴、共同缔造的强大合力。

　　我们坚信，有以习近平同志为核心的党中央亲切关怀，有省委、省政府和市委的坚强领导，有全市干部群众的共同努力，我们一定能够克服暂时困难，一定能够重塑沈阳辉煌，一定能够走出振兴发展新路，把沈阳老工业基地振兴伟大事业不断推向前进！

　　二、2017年重点工作安排

　　今年是党的十九大召开之年，是全面贯彻落实市第十三次党代会精神的开局之年，是扎实推进沈阳振兴发展的关键之年，做好今年政府各项工作意义重大。

　　今年，政府工作总体要求是：全面贯彻党的十八大和十八届三中、四中、五中、六中全会精神，认真落实习近平总书记系列重要讲话精神特别是关于东北振兴发展的重要指示批示精神，统筹推进“五位一体”总体布局，协调推进“四个全面”战略布局，认真落实“四个着力”要求，按照中央和省委经济工作会议部署，深刻把握经济发展进入新常态的重大判断，深刻把握以新发展理念为指导、以供给侧结构性改革为主线的政策框架，深刻把握稳中求进工作总基调这个治国理政的重要原则，全面落实市第十三次党代会确定的目标和任务，切实抓好稳增长、促改革、调结构、惠民生、防风险各项工作，促进经济平稳健康发展和社会和谐稳定。

　　今年全市经济社会发展的主要预期目标是：地区生产总值增长7%；固定资产投资增长15%；一般公共预算收入增长5%；社会消费品零售总额增长10%；城镇和农村居民人均可支配收入增长与经济增长同步；城镇登记失业率控制在3.7%以内。

　　经济增长预期目标确定为7%，考虑了与“十三五”规划目标相衔接，与全面建成小康社会要求相适应；考虑了保障就业、改善民生对经济增长的需要，以及稳定和引导社会预期的需要；考虑了做实经济数据后增长潜力将得到释放，经济增长有一定恢复性空间。

　　从实现预期目标的主要支撑看：工业方面，占全市工业80%的三大千亿产业持续增长，汽车行业将有一系列新车型投放市场，机械装备骨干企业新增订货明显增加，农产品加工龙头企业规模不断扩大；全年预计新增规模以上企业200户左右，也将带来新增量。服务业方面，金融机构本外币存贷款余额持续增长，房地产市场交易保持活跃，物流、会展、电商等现代服务业快速发展。投资方面，已落实开复工亿元以上项目570个，当年预计完成投资1250亿元左右，一批储备项目也将落地开工。消费方面，居民收入稳步提高，消费预期比较稳定，旅游、文化、教育、体育、健康等消费热点持续升温。特别是营商环境持续改善将吸引更多的企业和投资，“大众创业、万众创新”将催生更多新的市场主体。只要我们加倍努力，就一定能够实现预期目标。

　　做好今年政府工作，要深入贯彻新发展理念，破解发展难题、筑牢发展基础、厚植发展优势，推动沈阳新一轮振兴发展迈出新步伐、取得新突破。第一，必须坚持以振兴发展战略规划为引领，加快实施国际化、区域一体化、城市空间优化、产业多元化、人的现代化五大战略，切实做到一张蓝图干到底。第二，必须坚持以供给侧结构性改革为主线，用好加减乘除“四法统筹”，着力振兴实体经济，着力补齐发展短板，着力提升供给结构的竞争力。第三，必须坚持以打造国际化营商环境为切入点，注重服务便利和效率优先，着力解决突出问题，不断创新体制机制，充分激发市场活力。第四，必须坚持以创新驱动为根本动力，深入推进全面创新改革试验和自主创新示范区建设，集聚创新资源，活跃创新要素，改善创新生态，加快构建经济发展新方式。第五，必须坚持以人民为中心的发展思想，推进共同缔造，改善公共服务，搞好基本民生、保障底线民生、解决热点民生，让老百姓的日子越来越好，烦心事闹心事越来越少。第六，必须坚持改善政府服务，转变政府职能，改进管理方式，努力建设法治政府、诚信政府、廉洁政府、服务型政府，切实提高政府公信力、执行力。

　　(一)坚持目标导向、问题切入，确保国际化营商环境建设取得明显成效

　　全面落实市委、市政府打造国际化营商环境意见，切实通过环境的整体改善来集聚发展要素，激发社会潜能，释放发展内生动力。

　　分类对标，综合评估。对照世界银行指标体系，引入第三方专业评估机构，查找我市在企业全生命周期服务方面存在的差距；对照发达国家和先进地区，查找我市在政务、市场、开放、要素、设施、社会、法治等营商环境方面存在的问题，明确改进方向，找准工作路径，制定改进措施。

　　重点突破，协同推进。深入开展政务服务窗口质量、乱收费乱罚款乱检查、垄断性服务行业涉企服务、政府和企业失信、企业正常跨区迁移难等五大专项整治行动，着力提升窗口单位工作人员服务意识、文明素质、业务能力，切实以政务环境建设带动各领域营商环境建设。建立协调联动、监督考核和社会广泛参与机制，树立人人都有责任、人人都是环境的理念，形成共同缔造营商环境的强大合力，确保营商环境建设取得实效。

　　严格整改，全面提升。瞄准2020年营商环境排名进入前50的目标，结合评估报告建立问题清单，以“零容忍”态度抓紧整改百姓反映集中、企业反映强烈、社会反映普遍的突出问题。动员社会参与，加强社会监督，敢于揭短亮丑，确保整改到位。加强制度设计，建立长效机制，坚决防止问题反复反弹。积极宣传先进典型和工作推进成效，迅速改变沈阳营商环境形象。

　　(二)深化供给侧结构性改革，全力保持经济平稳健康发展

　　坚持以提高供给结构竞争力为主攻方向，突出重点任务，进一步发挥有效投资关键作用和实体经济支撑作用，推动全市经济提质增效。

　　深入推进“三去一降一补”。在去产能方面，支持企业技术改造、兼并重组，主动对接国家多边产能合作机制，通过海外建厂、工程承包等方式“走出去”，创新企业大型成套装备营销方式，努力拓展市场，提高企业生产负荷。在去库存方面，主要化解房地产库存。坚持分类施策，针对商业地产，重点是向文化、旅游、养老等业态和住宅用途转化；针对中心城区和县域商品住房，把好土地供应“闸门”，继续化解县域库存，确保中心城区去化周期和房价稳定在合理区间。在去杠杆方面，通过股权融资、债转股、资产重组等方式，拓宽直接融资渠道，帮助企业特别是国有企业降低负债率；规范政府举债行为、强化债务限额及预算管理，通过债券置换化解存量债务，通过政府与社会资本合作减少新增债务，有效防控政府性债务风险。在降成本方面，努力降低企业水电气暖、物流、融资等经营性成本，降低税费及审批等制度性成本，扩大“零审批”“零收费”园区试点。在补短板方面，通过发展新产业、新业态、新产品，补齐新经济短板；通过提升县域公共服务、培育壮大主导产业，补齐县域经济短板；通过完善政策支撑和服务体系，补齐民营经济短板。

　　大力实施项目带动战略。加快推进重大项目开复工，集中力量抓好华晨宝马第七代5系、米拉国际汽车研发与检测中心、利源铝型材深加工、盛京金融广场、沈阳铁路综合货场等433个重点产业项目，地铁、城市综合管廊等135个基础设施项目，垃圾及污水处理等48个生态环保项目，棚户区改造等55个民生项目，电网改造等30个能源项目。加强重大项目储备，深入研究投资需求，围绕培育新动能、发展新经济、延展产业链引进一批产业项目，围绕促进区域一体化、提升城市承载力、补齐民生短板策划一批基础设施、生态环保、改善民生项目，围绕优化城市空间做好东塔机场等一批重大搬迁改造项目前期工作，充实项目储备库。加快投融资体制改革，发挥政府投资引导作用，着力激发民间资本投资活力，加快实施一批政府与社会资本合作项目。有效破解项目建设难题，落实项目推进责任制，全力做好项目基础设施和公共服务配套，着力解决制约项目建设的土地征收和房屋拆迁等问题。

　　着力振兴实体经济。开展新动能培育行动，积极发展云计算、物联网、移动互联网等新业态，支持传统产业引入新技术、新机制、新模式，加快新旧动能转换。开展质量品牌提升行动，落实重点工业优质产品补贴政策，支持企业提高产品技术、工艺装备、能效管理水平，切实增加高质量产品和服务供给，培育一批“沈阳”品牌。开展企业规模升级行动，对重点产值增量企业、产品订单增长企业给予政策支持，建立中小企业入规升级培育库，实行“小升规”奖励制度。开展“工业强基”行动，依托重点工程、重大项目和骨干企业，发展本市需求量大、有市场、有技术支撑的“四基”产品，培育“专精尖”配套企业，不断提升本地基础产业的协作配套能力。

　　(三)深入推进改革开放创新，不断激发发展动力活力

　　大力实施创新驱动发展战略和国际化战略，深入推进全面创新改革试验，持续释放发展新动能。

　　全面推进“多规合一”改革。创新规划管理体制机制，建立部门业务联动、监控考核、动态更新等制度，确保“多规合一”有效落实。建立统一的空间规划体系，做到发展目标、空间坐标和用地指标“三标”合一，抓好城市总体规划、经济社会发展规划、土地利用规划、环境保护规划“四规”统筹，实现一张蓝图、一个平台、一张表单、一套机制，切实发挥规划在城市发展中的主导作用。

　　加快国资国企改革。优化国有资产布局，放大国有资本功能，推进国有资产集中统一监管，分类实施监管、分类推进改革、分类促进发展。改组设立国有资本投资运营公司，组建金控集团、城投集团、产投集团，搭建资产管理和处置平台，推动融资平台市场化改革，壮大产业引导基金规模。深化国有企业改革，完善国有企业法人治理结构，优化国有企业股权结构、组织结构、资产结构和产品结构，进一步健全航空、旅游、环保、养老等7大产业集团运转机制。推进混合所有制改革试点。加强国有企业党的建设，切实发挥企业党组织在企业改革发展、重大决策、人事任免等方面的重要作用。推进驻沈央企“三供一业”移交，分离企业办社会职能，积极解决厂办大集体等历史遗留问题。

　　深化行政体制改革。优化政府职能配置，深入推进“放管服”改革，严格执行政府清单管理制度，在中德装备园试点企业投资项目承诺制。复制厦门行政审批制度改革模式，推进职能整合、流程再造，推广“一个窗口受理、一站式办理、一条龙服务”，确保所有行政审批事项办结时限不超过国家法定时限的40%。深化商事制度改革，实行企业登记全程电子化和简易注销登记改革，全面推行企业“五证合一、一照一码”、个体工商户“两证合一”和“双随机、一公开”监管，建立信用公共信息共享平台，充分激发市场主体活力。稳步推进事业单位分类改革。

　　大力发展民营经济。完善融资服务体系，加强担保体系建设，运用助保贷、融资扶持基金、流动性债权基金、应收账款质押和股权质押贷款等金融工具，缓解企业融资难、“过桥难”等问题。完善创新创业服务体系，建设一批中小微企业创业基地、公共技术服务平台和成果孵化器，开展“互联网+小微企业”专项行动，提升中小微企业创新能力。完善市场服务体系，组织中小微企业参加贸易展示洽谈，支持中小企业与大企业协作配套，鼓励优势民营企业“走出去”，申办“2018APEC中小企业技术交流暨展览会”。完善人才服务体系，开展科研院所与中小企业服务对接，通过高等院校、职业技术学校培养一批技能人才。引导民营企业建立现代企业制度，支持优秀民营企业上市。

　　积极推进科技创新。加快国家自主创新示范区建设，构建具有沈阳特色的创新体制机制，充分释放政策红利，打造良好创新生态。努力提升创新能力，深化科技计划改革，引导企业加大研发投入，对接京沈高校、科研院所开展100项重大技术联合攻关。支持企业设立海外研发机构，并购海外研发团队。实施科技成果转化工程，持续跟踪支持一批重大研发项目熟化、转化。遴选200家企业实施高新技术企业培育计划，实施科技“小巨人”成长计划。加快创新平台建设，大力培育IC装备等38个产业技术创新联盟，引进建立与我市产业密切关联的院士工作站、产业技术研究院、工程研究中心和技术转移中心，围绕机器人、数字医疗等行业龙头企业推进全产业链创新布局，提高协同创新水平。完善创新服务体系，积极推动科研院所、高等院校开放资源，搭建工业设计、检验检测、科学数据、大型科学仪器设备共享等公共服务平台。加强知识产权保护、运用，创建知识产权示范市，开展知识产权质押融资试点，为创新型企业搭建融资平台。深入推进“盛京人才”战略，实施盛京优才集聚计划、盛京英才支持计划和重点产业高层次紧缺人才奖励计划，培养集聚各领域领军人才、管理人才、专业技能人才，实行人才引进培养、服务保障特殊政策，争创老工业基地人才管理改革试验区。

　　实施更加积极的开放战略。借鉴厦门模式，以推进负面清单投资管理、贸易便利化和金融开放为核心，加快自贸区改革创新。以中德装备园为载体，抓好对德国以及欧洲各国的招商引资、企业并购，促进中德企业扩大技术、管理、双元制教育合作，办好德国企业沈阳行活动。实施精准招商，围绕壮大汽车、装备制造等千亿产业集群加强产业链招商，围绕发展生物医药、新材料、新能源、大数据、电子商务等新产业、新业态加强新经济招商，盯住长三角、珠三角、京津冀等创新活跃地区加强科技成果转化项目招商。积极融入“一带一路”，优化口岸通关环境，打造“沈阳港”和空港经济区等开放枢纽，探索“沈满欧”、“沈满俄”班列长效运营机制。深化友城合作，拓展对外交流渠道。加强对口合作，以项目清单为抓手，建立与北京、江苏、厦门合作机制，实现互利互惠、共赢发展。

　　(四)加快推进产业转型，着力构建现代产业体系

　　大力实施产业多元化战略，抓龙头、搭平台、铸链条，加快构建“五大板块”协调发展的现代产业体系。

　　加快推进工业转型升级。发挥先进装备制造业引领作用，推动制造业智能化、高端化、服务化，对接德国“工业4.0”，推进“中国制造2025”试点示范城市建设，继续开展制造业智能升级行动，建设装备制造业大数据中心，支持企业打造智能工厂、智能车间；鼓励骨干企业加大科技投入，加快产品结构升级，提升高端装备发展水平；加快实施沈阳机床i5战略计划，促进i5智能机床在重点领域的推广应用，支持特变沈变、北方重工等企业承揽“交钥匙”工程。推动新兴产业规模化，开工建设50个新兴产业、25个新兴业态项目，争取庞巴迪Q400飞机总装项目落地，力争机器人产业产值突破90亿元。推动优势产业集群化，以龙头企业为核心，打造航空、输变电装备、机器人、新一代信息技术等千亿产业链(集群)。推动军民融合发展，启动航天十二院军民融合技术转移中心项目，支持新松建设军民融合特色园区，争创国家军民融合创新示范区。

　　大力发展都市型现代服务业。着力发展与国际化城市功能相匹配的生产性服务业。积极推进金融创新，加快集聚新型金融机构和培育区域性资本市场、要素市场，推动金融商贸开发区晋升国家级开发区。做优做强现代物流业，强化物流通道拓展、物流设施建设和物流技术应用，加快跨境电商等物流产业园建设，推动快递业与电子商务联动发展，打造“一带一路”重要节点城市。加快建设东北亚会展中心城市，办好中国装备制造业博览会、国际飞行大会等大型会展活动。着力发展与都市人群多样化需求相适应的生活性服务业。加快培育壮大旅游、文化、体育、健康、养老等“幸福产业”，推进国有景区改革，发展工业、乡村、清文化等主题旅游，推动盛京皇城创建国家5A级旅游景区。着力促进传统商业向“线上线下”结合、“体验销售”结合转变，改善中街、太原街、长江街等商业街区消费环境，让一批老街、老店、老市场重新焕发活力。

　　做优做精“三高”型都市农业。深化农业供给侧结构性改革，继续优化种植业结构，促进一二三产业融合发展，大力实施农产品品牌战略，提高农业综合效益，促进农民增收。建设都市农业产业体系，加快环城、沈康、沈阜等示范带和蒲河经济带建设，打造一批高端精品、“名特优新”农产品基地和休闲观光农业基地，新增10户农业产业化龙头企业，提高农产品精深加工水平。完善都市农业生产体系，加强农业基础设施建设和粮油高产示范区建设。稳定粮食生产，扩大肉、蛋、奶产量，保障“菜篮子”安全，支持寒富苹果等特色农产品开拓市场。培育都市农业经营体系，基本完成农村土地承包经营权确权登记，推进“三权分置”改革，促进适度规模经营。加强新型职业农民技能培训，新增500家农民合作社、230个家庭农场。推进供销社改革，搭建涉农电商平台，建立新型高效的农产品流通网络。

　　(五)加快推进城市转型，切实提升城市承载力和宜居度

　　坚持内涵式发展，正确处理规模与效益、建设与管理、主城与副城的关系，优化发展空间，完善城市功能，增强城市魅力，努力建设北国风光宜居家园。

　　提升城市统筹规划建设水平。按照“一河两岸、一主三副”的规划布局，大力推进基础设施和教育、医疗等公共服务资源向浑南新城区布局、向副城延伸，畅通新城与老城、主城与副城之间的交通联络。统筹城乡规划建设，支持苏家屯区开展市场化推进产城融合综合试点、辽中区和新民市开展国家新型城镇化综合试点、康平县建设城乡统筹发展示范区，加强县城、重点镇、特色镇建设，加快以县为单元的就地城镇化。统筹区域发展，积极参与沈抚新区建设，完善沈阳—营口陆海联运系统，推进铁岭—沈阳—营口城际铁路建设，打造沈阳经济区重要发展轴。

　　提升基础设施现代化水平。完善城市轨道交通系统，开工建设地铁2号线南延线、3号线和6号线，完成1号线东延线和4号线北延线前期工作。完善城市快速交通系统，建设东一环、长青街、沈辽路等快速路，确保南北快速干道、五爱互通立交桥全面投入使用，启动沈康高速三环连接线工程。实施西北连接线、中央大街跨河桥及二环快速路节点改造等工程，打通环路连接线和重要交通节点。加快发展城市慢行系统，建设高品质专用绿道。结合新城建设和老城改造，实施国省干道提标升级工程，加快推进南运河等城市综合管廊建设，启动沈河东部新城“海绵城市”示范区建设，实施西部、东部电力扩容工程。

　　提升城市智慧化水平。进一步扩大4G无线网络、宽带网络、免费智能WiFi覆盖范围，打造宽带乡村、光网城市和无线城市。继续推进智慧交通、智慧医疗、智慧社区等各类惠民项目，提升市民对智慧沈阳建设的获得感。完善智慧沈阳统一平台和数据安全保障体系，实现大数据共享、开放与安全相互促进。扎实推进国家大数据综合试验区建设，组建大数据交易平台机构，形成数据交易机制，建设集存储、处理、运营、安全及标准于一体的东北区域大数据中心。依托华录集团、国信优易、树根互联、清华大学工业大数据研究中心等企业和机构，推进铁西国家信息中心大数据创新创业(沈阳)基地、浑南国家大学科技城、沈北国际(中韩)创新产业园等大数据领域的基地、园区和项目建设，加快大数据产业布局，开展工业大数据应用试点示范。承办好第四届中国智慧城市(国际)创新大会。

　　提升城乡宜居水平。严格执行生态保护红线制度，保育“三环三带四楔”生态廊道。促进城乡绿化提档升级，改造辽河七星湿地、南塔等城市公园，提升城区主路绿化景观，加快退耕还林、荒地造林。持续推进蓝天工程，继续淘汰治理黄标车，淘汰拆除600台燃煤锅炉，加强秸秆资源化利用，努力增加大气优良天数。加快推进全域河湖水系连通工程，实施北部、沈水湾等4座污水处理厂提标改造及南部污水处理厂二期建设等工程，综合治理细河、白塔堡河、小南河等黑臭水体，推进卧龙湖、珍珠湖生态工程建设，打造亲水环境。完善城市生活垃圾分类回收管理机制，确保老虎冲餐厨废弃物处理场投入运行。加快推进老虎冲、大辛、张士垃圾焚烧发电项目建设，实施祝家150万吨积存污泥无害化处理和污染源在线监控项目。加大宜居乡村建设力度，开展99个宜居示范村镇创建。实施全民节能行动计划，加快国家循环经济示范城市建设。

　　提升城市管理精细化水平。深入推进城市管理和执法体制改革，建立形成制度化、规范化、科学化、智能化城市管理工作机制，建立城市精细化管理平台，实现城市管理全方位覆盖、全时段监管、高效能运转。着力解决百姓身边问题，将精细化管理向小街小巷、小区周边延伸，向市政设施维护、环卫作业、园林绿化、市容景观提升拓展。实施居民住宅区物业提升工程，加强老旧住宅区综合改造后的规范化管理。加强交通疏导，完善重点地区指路标志和流量检测系统，加大各类停车场建设力度，强化小街小巷静态交通治理。加快创建“公交都市”，新开调整30条公交线路，新增更新新能源、清洁能源公交车1350台，缩短高峰期车隔，不断提高公交舒适度和便捷度。

　　(六)加快推进社会转型，共同缔造幸福沈阳

　　创新社会治理体系，转变社会治理模式，织牢织密民生保障网，不断提升人民群众生活质量和水平。

　　着力推进社会治理创新。以社区为基本单元，努力构建“纵向到底、横向到边、协商共治”的社会治理体系，把权力下放到基层、将服务推进到基层。加快和谐社区建设，提升市民文明素质，建立健全居民代表会议制度，引领居民“协商共治”，增强居民对社区的认同感。建设社会组织孵化基地，加大政府购买服务力度，引导社会组织参与社会治理、提供公共服务。加强双拥共建，促进军政、军民团结。强化市民主人翁意识，塑造城市共同精神，实现“决策共谋、发展共建、建设共管、效果共评、成果共享”。

　　加大就业和社会保障力度。坚持就业优先战略，多渠道开发就业岗位，实施万人创业工程，加强大学生、复转军人等重点人员就业服务，新增就业8.8万人以上。推进城乡居民基本医疗保险一体化，进一步提高企业退休人员养老待遇水平，确保养老金及时足额发放。加快完善社会化养老服务体系，推广医养结合新型养老模式，推进居家和社区养老服务改革试点，新增区域性居家养老服务中心20个、日间照料站30个、养老床位4000张。提高困难群体救助标准，逐步缩小城乡救助差距。健全精准扶贫长效机制，继续推进区县结对帮扶共建，实现行政村通客运班车率、饮水安全受益率、有线电视覆盖率、新农合参合补助率四个100%，坚决打赢脱贫攻坚战。大力发展残疾人事业、社会福利事业和慈善事业，开发应用盲人“乘车易”，加强困境儿童监护、生活、教育、医疗等方面保障，为全市孤儿和残疾人投保意外伤害险。继续办好一批民生实事，改造80个老旧小区、1.2万户(套)棚户区、805户农村危房，完善40个新建小区配套，推进111个小区、5.5万户老旧住宅“三网”配套改造，力争通过社会化方式完成298台老旧电梯维修更新，基本建成1万套保障房，实施300万平方米节能暖房工程，进一步提高供暖质量。

　　不断提升公共服务水平。促进教育公平，提高教育质量，新增公办幼儿园学位3500个，改造农村义务教育薄弱学校100所，完成全市中小学卫生保健所达标改造，完善中小学实验与运动器材配备，推动职业教育、高等教育与地方经济融合发展。推进老年人大学向社区延伸，不断完善终身教育体系。积极创建国家卫生城市和健康城市。适时启动城市公立医院综合改革，加强医联体建设，促进分级诊疗、多点执业。加快中国医大一院浑南国际医院、东北国际医院、沈阳积水潭医院建设，推进市中医院、口腔医院等重点医院迁建工程，完成市职工康复医院改造工程。繁荣发展文化事业，鼓励艺术精品创作，加强国际文化艺术交流与合作，开展“文化下乡”、流动博物馆送展等文化惠民活动。实施“九·一八”历史博物馆改造，确保市少儿图书馆迁建工程一期投入使用，市青少年宫、妇女会馆、艺术学校等项目按期完工。鼓励利用老厂房、老街区建设文创产业园区，培育新型文化业态和特色文创企业，做强国家文化产业示范基地，切实提高城市文化软实力。大力发展体育事业，推进公共体育场馆向市民免费、低收费开放，启动棋盘山户外全民健身中心项目建设，办好市第十四届全民运动会、国际市民体育联盟首届市民冬奥会等大型体育赛事，打造足球城市品牌，建设体育强市。

　　深化平安沈阳建设。完善立体化社会治安防控体系，依法严厉打击各类违法犯罪。建立多元化的群众诉求表达渠道，落实信访工作责任制，依法分类解决信访问题。完善食品药品可追溯体系和检验检测体系，严厉打击食品药品领域违法行为，扎实推进国家食品安全城市创建工作。推进安全生产领域改革发展，严格实行安全生产党政同责、一岗双责、齐抓共管、失职追责，加强安全管理制度化、标准化、网格化、信息化建设，坚决防范重特大安全事故发生。做好气象灾害预报预警，完善应急和防灾减灾救灾机制，增强应急救援能力，保障群众安居乐业、社会安定有序。

　　(七)不断加强政府自身建设，努力提高施政能力和服务水平

　　实现全面振兴对政府自身建设提出了更高的要求，必须进一步转变政府职能，不断提高政府工作质量和效率。

　　坚持正确政治方向。牢固树立“四个意识”特别是核心意识、看齐意识，认真落实全面从严治党要求和“讲诚信、懂规矩、守纪律”要求，深刻吸取辽宁拉票贿选案教训，切实在思想上政治上行动上始终同以习近平同志为核心的党中央保持高度一致，切实把党中央国务院、省委省政府和市委各项决策部署落到实处。

　　扎实推进依法行政。自觉接受人大依法监督、政协民主监督和社会广泛监督，严格依法决策、依法行政，努力建设法治政府。完善政府守信践诺机制，建立健全政务和行政承诺考核制度，严格履行各项政策承诺,努力建设诚信政府。切实强化党风廉政建设主体责任，全面落实“一岗双责”，认真落实中央八项规定精神，积极构建“亲”“清”新型政商关系，努力建设廉洁政府。强化政府公共服务职能，提高服务能力和服务效率，推广窗口单位预约服务、延时服务，努力建设服务型政府。

　　持续加强作风建设。坚持问计于民、问需于民，听取政府参事、顾问和智库专家意见，深入开展调查研究，落实民主集中制，促进政府决策科学化。发挥“关键少数”示范带动作用，自觉践行“三严三实”，以上率下，团结协作，形成干事创业的浓厚氛围。进一步紧张起来，扎实细致，快速反应，敢于担当，善于担当，雷厉风行抓落实。建立健全容错机制，坚持“三个区分开来”，旗帜鲜明地为敢于担当的干部担当，为敢于负责的干部负责，让敢担当、有作为的干部没有后顾之忧。

　　各位代表，沈阳已经迈上了振兴发展的新征程。让我们更加紧密地团结在以习近平同志为核心的党中央周围，在市委的坚强领导下，坚定信心、凝心聚力、开拓进取、扎实工作，奋力开创振兴发展新局面，以优异成绩迎接党的十九大胜利召开！
